# Automatic optic disc detection in colour fundus images by means of multispectral analysis and information content

**DOI:** 10.7717/peerj.7119

**Published:** 2019-06-27

**Authors:** M. Elena Martinez-Perez, Nicholas Witt, Kim H. Parker, Alun D. Hughes, Simon A.M. Thom

**Affiliations:** 1 Institute of Research on Applied Mathematics and Systems, Department of Computer Science, Universidad Nacional Autónoma de México, Mexico City, Mexico; 2 National Heart & Lung Institute, Imperial College, London, UK; 3 Department of Bioengineering, Imperial College, London, UK; 4 Institute of Cardiovascular Sciences, University College London, London, UK

**Keywords:** Optic disc detection, Visual multispectral imaging, Information content, Retinal images, Computer-based systems

## Abstract

The optic disc (OD) in retinal fundus images is widely used as a reference in computer-based systems for the measurement of the severity of retinal disease. A number of algorithms have been published in the past 5 years to locate and measure the OD in digital fundus images. Our proposed algorithm, automatically: (i) uses the three channels (RGB) of the digital colour image to locate the region of interest (ROI) where the OD lies, (ii) measures the Shannon information content per channel in the ROI, to decide which channel is most appropriate for searching for the OD centre using the circular Hough transform. A series of evaluations were undertaken to test our hypothesis that using the three channels gives a better performance than a single channel. Three different databases were used for evaluation purposes with a total of 2,371 colour images giving a misdetection error of 3% in the localisation of the centre of the OD. We find that the area determined by our algorithm which assumes that the OD is circular, is similar to that found by other algorithms that detected the shape of the OD. Five metrics were measured for comparison with other recent studies. Combining the two databases where expert delineation of the OD is available (1,240 images), the average results for our multispectral algorithm are: TPR = 0.879, FPR = 0.003, Accuracy = 0.994, Overlap = 80.6% and Dice index = 0.878.

## Introduction

The eye is a window to a microcirculatory bed that allows information to be captured in the domain of visible light, and is suitable for making non-invasive clinical diagnoses. Digital images of the fundus of the eye have important diagnostic and potential prognostic roles. For example, quantitative measures of the retinal microvasculature can identify abnormalities at a very early stage in the process of cardiovascular disease (Tapp et al., 2007, 2015; Wong, 2014) and predict ischemic heart diseases (Witt et al., 2006), cerebral infarction and white matter lesions (Wong, 2004; Hughes et al., 2016), cognitive decline (Wong et al., 2002), future hypertension (Ding et al., 2014) and renal disease (Wong et al., 2004) independently of conventional risk factors. However, quantitative assessment of the retinal microvasculature remains time-consuming and restricted to specialist research settings.

Over several years, our group has focused on the development of computer-based tools for the quantification of retinal microvascular branching parameters using digital colour and monochromatic images taken with conventional fundus cameras. These parameters have been measured using a semi-automatic grading system running on a personal computer.

**Figure 1:**
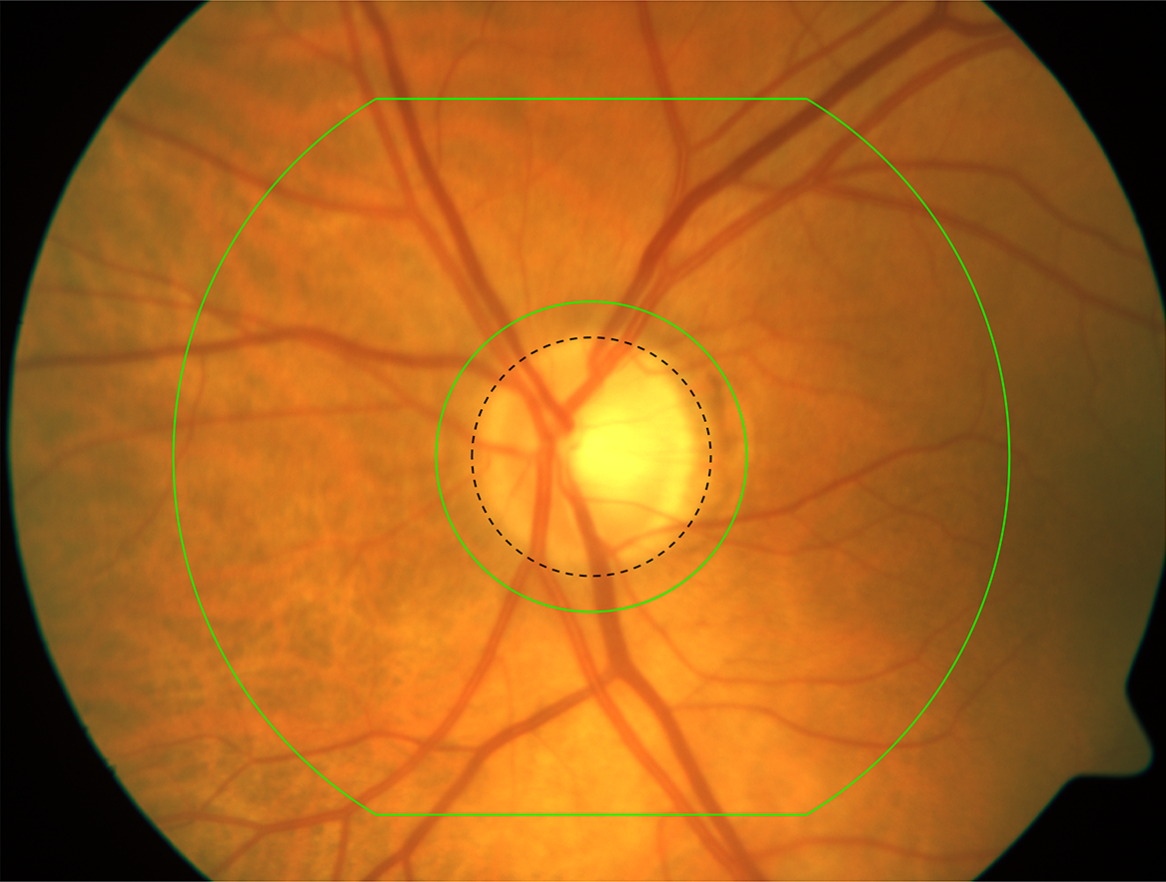
The grid used for measuring retinal images. The grid is manually centered on the optic disc of radius *R_a_* (black dashed circle). Measurements are made from bifurcations occurring between the inner the outer green boundaries derived from *R_a_*. *R_a_* is fixed a priori for the whole database.

Measurements are performed from the optic disc (OD) outwards, within a distance of 1.5 disc diameters from the disc boundary, guided by a reference grid, and include (i) arterial and venular diameters, (ii) arterial-to-venular ratio, (iii) arterial bifurcation angles, (iv) length/diameter ratios and (v) vessel tortuosity. A screenshot from the current system is shown in Fig. 1. The reproducibility of this technique has been reported previously (Chapman et al., 2001; Witt et al., 2010).

A high volume of scans necessitates a rapid, largely automated analysis which requires computer-aided image processing to achieve acceptable quantification. The detection of the centre of the OD in our current system is still a manual process. Its automatic detection is a fundamental step since it is used as a landmark for all successive measurements. We are thus interested in detecting the OD and determining its centre automatically. We also recognise that automatic detection of the OD location and delineation, that can be used for a wide variety of retinal images, may be of interest in other contexts.

The following introduction to fundus imaging is based on the excellent summary by the Ophthalmic Photographer’s Society (Bennett, 2011). The appearance of the vessels and other structures in the fundus changes according to the wavelength. The visible light spectrum is divided into short, intermediate and long wavelengths, which represent the primary colours: blue, green and red centred at 450, 540 and 625 nm, respectively. Digital cameras that acquire fundus images over a spectrum greater than that of visible light exist but are not yet in routine use (Zimmer et al., 2014).

The wavelength of the blue light increases the visibility of the anterior retinal layers, which are usually almost transparent in white light. This wavelength is absorbed by the pigmentation of the retina and blood vessels, providing a dark background against which the specular reflections and scattering in the anterior layers of the fundus are enhanced.

The wavelength of the green light is also absorbed by the blood but is partially reflected by the retinal pigmentation unlike the blue light. Green light provides excellent contrast and the best overall view of the fundus, improving the visibility of the retinal vasculature and other lesions such as haemorrhages, drusen and exudates. For this reason, photographs using green filter, also called ‘red-free’, are taken routinely and are commonly used for automatic detection of vessels (Martinez-Perez et al., 2007).

Retinal pigmentation appears clear and more transparent in the wavelength range of red light, revealing more of the choroidal pattern. The overall contrast with the background is considerably reduced with this wavelength, since many structures of the retina are red. The retinal vessels look lighter and are less obvious at longer wavelengths, with the arteries containing oxygenated haemoglobin appearing lighter compared to the veins which contain ∼40% de-oxygenated haemoglobin. The OD also appears very light and almost without distinctive features, often making this wavelength the most appropriate for the identification of the margins of the OD.

Digital sensors exhibit a more linear response compared to film-based imaging, but generally with a lower latitude of exposure. Excessive exposure can lead to saturation of the output from a digital sensor (most commonly in the red channel of retinal images), which is detrimental to the quality of the image in its brightest regions. Exposure control requires a delicate balance between flash output, sensor gain and gamma settings.

## Related Work

Although digital retinal photographs are taken routinely in colour, most of the studies using digital images for the location and/or delineation of the OD, use a single channel or a linear combination of channels. Reviewing the literature of the last 5 years, some use either the green channel (Abdullah, Fraz & Barman, 2016; Basit & Fraz, 2015; Abdullah & Fraz, 2015; Sigut et al., 2017; Marin et al., 2015; Roychowdhury et al., 2016; Giachetti, Ballerini & Trucco, 2014), the red channel (Mary et al., 2015), a combination of red and green channels (Morales et al., 2013; Dashtbozorg, Mendonça & Campilho, 2015; Lim et al., 2015), or some other colour representation model: such as CIELab (Yu et al., 2012; Zilly, Buhmann & Mahapatra, 2017), CIELuv (Welfer, Scharcanski & Marinho, 2013) and HSV (Cheng et al., 2013).

There are several algorithms published in the literature describing the detection and/or delineation of the OD. They can be grouped into three major categories: (i) those based on morphology and intensity values (Welfer, Scharcanski & Marinho, 2013; Morales et al., 2013; Marin et al., 2015; Roychowdhury et al., 2016; Sigut et al., 2017); (ii) model-based such as: grow-cut (Abdullah & Fraz, 2015; Abdullah, Fraz & Barman, 2016), watershed (Basit & Fraz, 2015), multiresolution filters (Dashtbozorg, Mendonça & Campilho, 2015), active contours and deformable models (Cheng et al., 2013; Giachetti, Ballerini & Trucco, 2014; Mary et al., 2015), level sets (Yu et al., 2012); or (iii) those based on artificial intelligence techniques, such as convolutional neural networks (Lim et al., 2015; Zilly, Buhmann & Mahapatra, 2017; Fu et al., 2018; Cheng et al., 2018).

If we rely on the spectral content of the fundus images described above, it would seem more suitable to search for the OD in the red channel, since there are fewer structures visible in the retina and the OD appears better contrasted. However, in practice a large proportion of images are saturated in the red channel, usually due to overexposure. This often makes it impossible to detect the OD in the red channel.

We propose a fast, fairly simple algorithm to find the OD and its centre, that exploits all the spectral information in the colour digital image to overcome the problem of saturation. It combines: (i) using all the spectral information present in the three channels (RGB) of the digital colour image to locate the region of interest (ROI) where the OD lies, (ii) measuring the Shannon’s information content per channel in the ROI, to decide which channel is most appropriate for searching for the OD centre using the circular Hough transform (CHT).

The major contribution of this article is to enable analysis of a wide range of images taken from diverse samples under different resolutions, fields of view (FOV) and illumination conditions. By using all the spectral colour information, we are able to locate the centre of the OD in a robust and simple way even in the majority of overexposed images. By using the CHT we are also able to find an approximation of the delineation of the OD, which may be useful for other applications. Our results are competitive with other state-of-the-art methodologies with a low percentage of detection error.

## Materials

Three different databases are used for evaluation, one local and two public. A total of 2,371 colour images are analysed and used for evaluation purposes.

### SABRE (local database)

Southall and Brent revisited (SABRE) (Hughes et al., 2016) is a tri-ethnic cohort (Europeans, South Asians and African Caribbeans) in London, UK (The ethical approval for the study was obtained from the Imperial College London, St Mary’s Research Ethics Committee. REC reference 07/H0712/109. Written, informed consent was obtained from each participant). It is a community-based sample of men and women aged between 40 and 69 who were recruited between 1988 and 1990. Participants were excluded from retinal photography if they had glaucoma or any condition that prevented adequate imaging of the retina. The fundus of both eyes was imaged using a Zeiss FF450+ fundus camera (30° field) with an Allied Vision Tech Oscar 510C CCD (2,588 × 1,958 pixels). Digital retinal photography was performed on 1,205 individuals and analysable images for retinopathy grading and quantitative microvascular analysis were obtained in 1,131 individuals (aged 69 ± 6y; 77% male; 37% South Asian, 15% African Caribbean). These are the images considered for evaluation in this work. Quantitative retinal vessel measurements have been made using a semi-automated program (Chapman et al., 2001). OD centres were hand marked by the graders during the grading process, this information was saved in the configuration and measurement files.

### DRIVE (public database)

The DRIVE database (Staal et al., 2004) consists of 40 images which were captured in digital form using a Canon CR5 non-mydriatic 3CCD camera with a 45° field of view (FOV). The images are of size 768 × 584 pixels, 8 bits per colour channel. The images are classified by the authors into test and train sets with 20 images each. The OD boundary was hand-delimited in both sets by one of the authors (MEM). We used the 40 images without distinction.

### MESSIDOR (public database)

The MESSIDOR database (Decencière et al., 2014) consists of 1,200 images acquired in three different ophthalmology departments using a 3CCD colour video camera on a Topcon TRC NW6 non-mydriatic retinograph with a 45° FOV and three different resolutions: 1,440 × 960, 2,240 × 1,488 and 2,304 × 1,536 pixels. The OD boundary of all images were manually delimited by experts at the University of Huelva (2017).

## Methods

The method is carried out in two phases: (i) using all the spectral information present in the three channels (RGB) of the digital colour image to locate the ROI where the OD lies, (ii) measuring the Shannon information content per channel in the ROI, to decide which channel is most appropriate for searching for the OD centre using the CHT. Our hypothesis is that using all the colour information in a retinal image will lead to better results than using information from a single channel. We test this hypothesis by comparing results of our multispectral analysis (MS) to identical analyses using only the green (G) or only the red (R) channel.

The SABRE images are used as the examples in the description of the algorithm; its application to the other databases is straightforward. The only parameter in our algorithm that must be known a priori is the approximate size of the OD diameter in pixels in the database. This parameter is called the nominal radius, *R_a_*. Assuming as a prior that the OD diameter is 1.75 mm (Ramrattan et al., 1999) this can be converted to pixels based on the individual camera used. It depends on the settings used for the fundus camera: particularly the spatial resolution (in pixels) with which the images were acquired and the FOV (magnification factor) used. In the case of SABRE images, this information is contained in the configuration file, and it is set, for the whole database before the grading is initiated. For the case of the other two databases, it is estimated by manual measurement of one image selected at random from the data set.

### Determine the ROI (coarse scaling)

#### Pre-processing

The colour images of the SABRE database are 2,588 × 1,958 pixels. To locate the OD, which is one of the largest objects in the image, it is not necessary to use this degree of spatial resolution. As part of the algorithm all images that have one dimension larger than 1,000 pixels are reduced to ¼ of their original size using a bicubic interpolation method, solely to reduce the processing time. The rest of the process described below is done on the reduced colour image versions.

A binary mask is applied to the image in each of the channels, with the aim of processing only the values contained within the FOV. The binary mask is obtained by using a single channel (e.g., red) and executing the Otsu algorithm for two classes (Otsu, 1979). To avoid cases where the FOV circle is not complete, the major axis of the binary object is obtained and used to fit the circle with the major axis.

**Figure 2:**
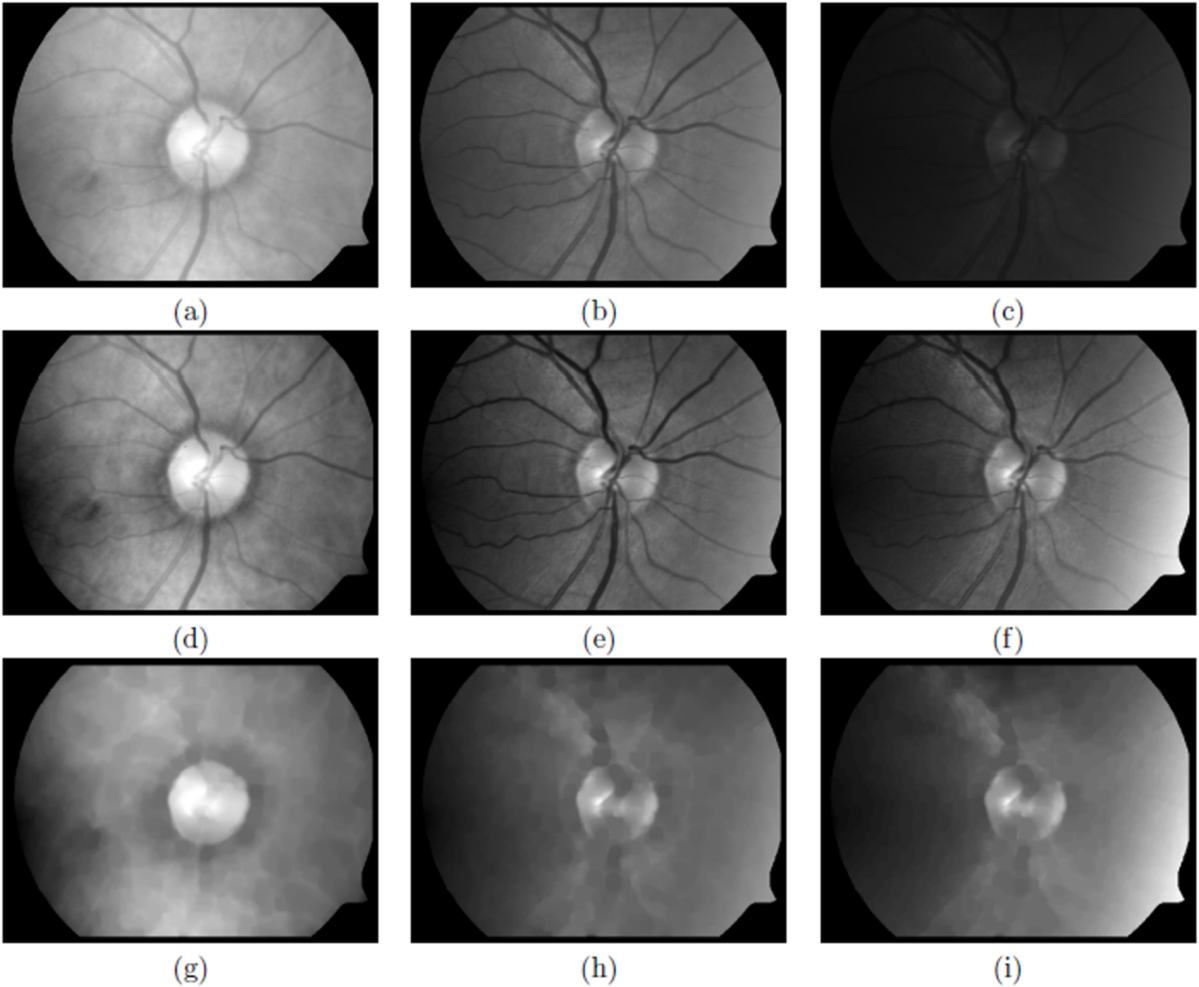
An unsaturated image. (A–C) Original red, green and blue channels, respectively, (D–F) normalised red, green and blue channels, respectively and (G–I) closing operation to filter blood vessels for red, green and blue channels, respectively.

**Figure 3:**
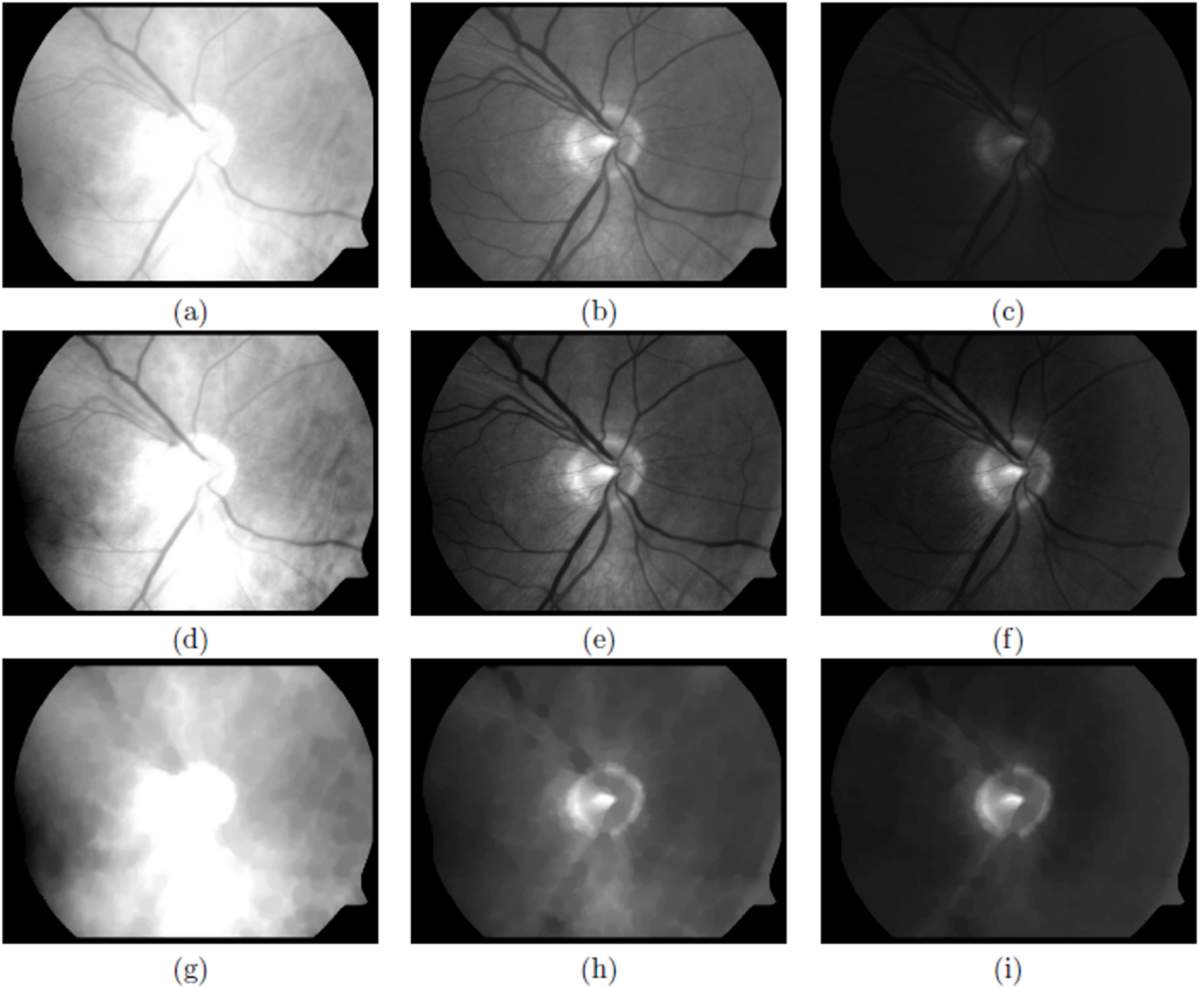
A saturated image. (A–C) Original red, green and blue channels, respectively, (D–F) normalised red, green and blue channels, respectively and (G–I) closing operation to filter blood vessels for red, green and blue channels, respectively.

The masked image will be referred to as the ‘image’ in the following. Figure 2 shows an example of an unsaturated and Fig. 3 a saturated image.

To increase the contrast in each of the channels, a basic transformation is applied that adapts the dynamic range of the input image to the whole spectrum of quantisation values:

(1) $${I_{{\rm{out}}}} = {{{I_{{\rm{in}}}} - {\rm{min}}\left( I \right)} \over {\max \left( I \right) - {\rm{min}}\left( I \right)}};$$

with min(*I*) and max(*I*) the dynamic range of the input image, *I*_in_ (See Figs. 2D–2F and 3D–3F).

A gaussian filter, *g*, is applied to each of the channels. The size of the filter is equal to the nominal radius *R_a_*.

(2) $$g\left( {x,y} \right) = {1 \over {2{\rm{\pi }}{{\rm{\sigma }}^2}}}{e^{{{ - \left( {{x^2} + {y^2}} \right)} \over {2{{\rm{\sigma }}^2}}}}};$$

with $x,y \in \left[ { - {R_a}/2,{R_a}/2} \right]$, where we found that σ = *R_a_*/4, gives adequate results.

**Figure 4:**
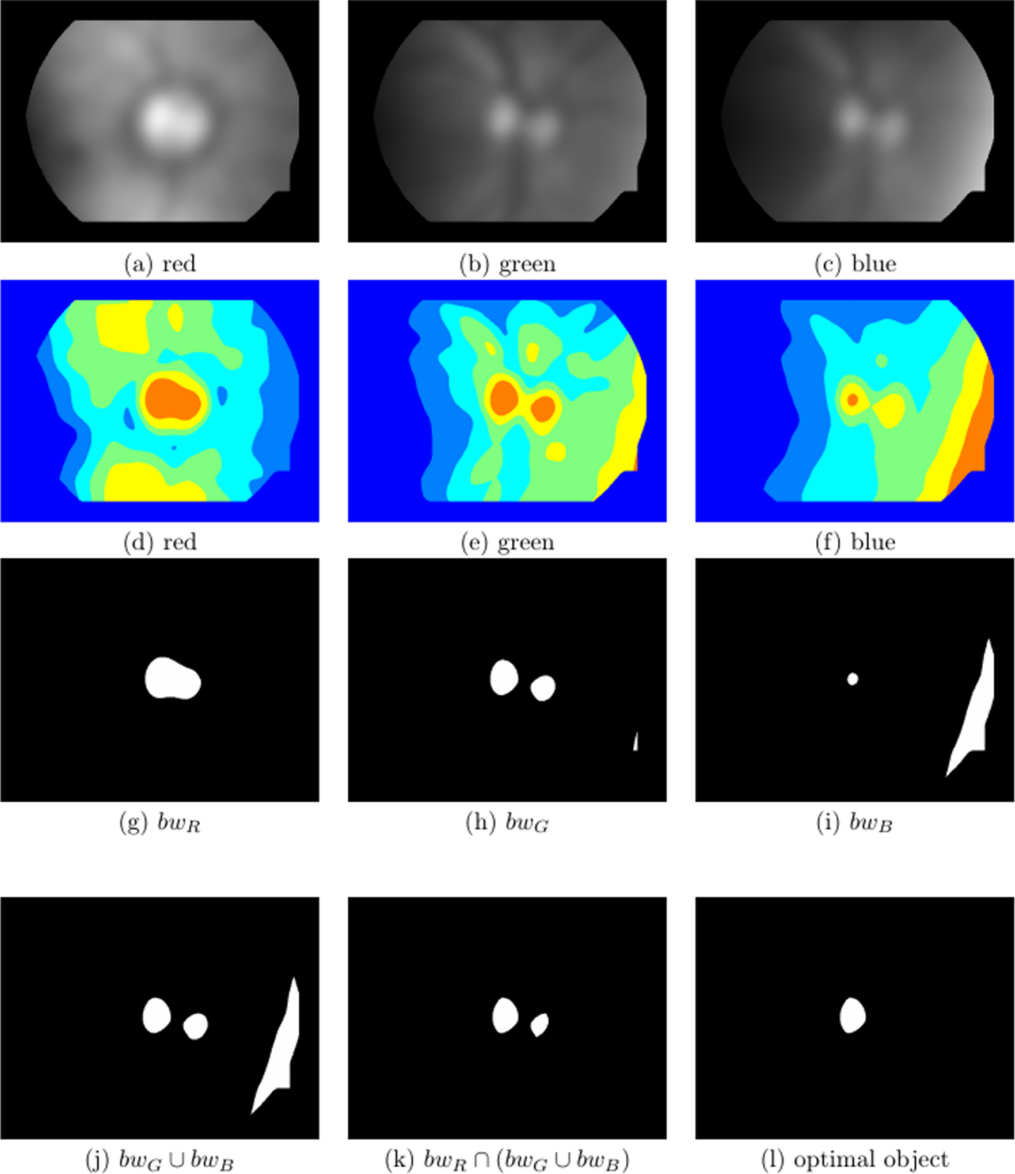
Visual multispectral analysis of an unsaturated image. (A–C) Normalised and Gaussian filtered images from the red, green and blue channels, respectively, (D–F) show the classes after multithreshold processing from the image above, for *k* = 5, (G–I) are the maximum class defined as bw_R_, bw_G_ and bw_B_, respectively, (J) bw_G_ ∪ bw_B_, (K) bw_R_ ∩ (bw_G_ ∪ bw_B_) and (L) optimal selected object.

**Figure 5:**
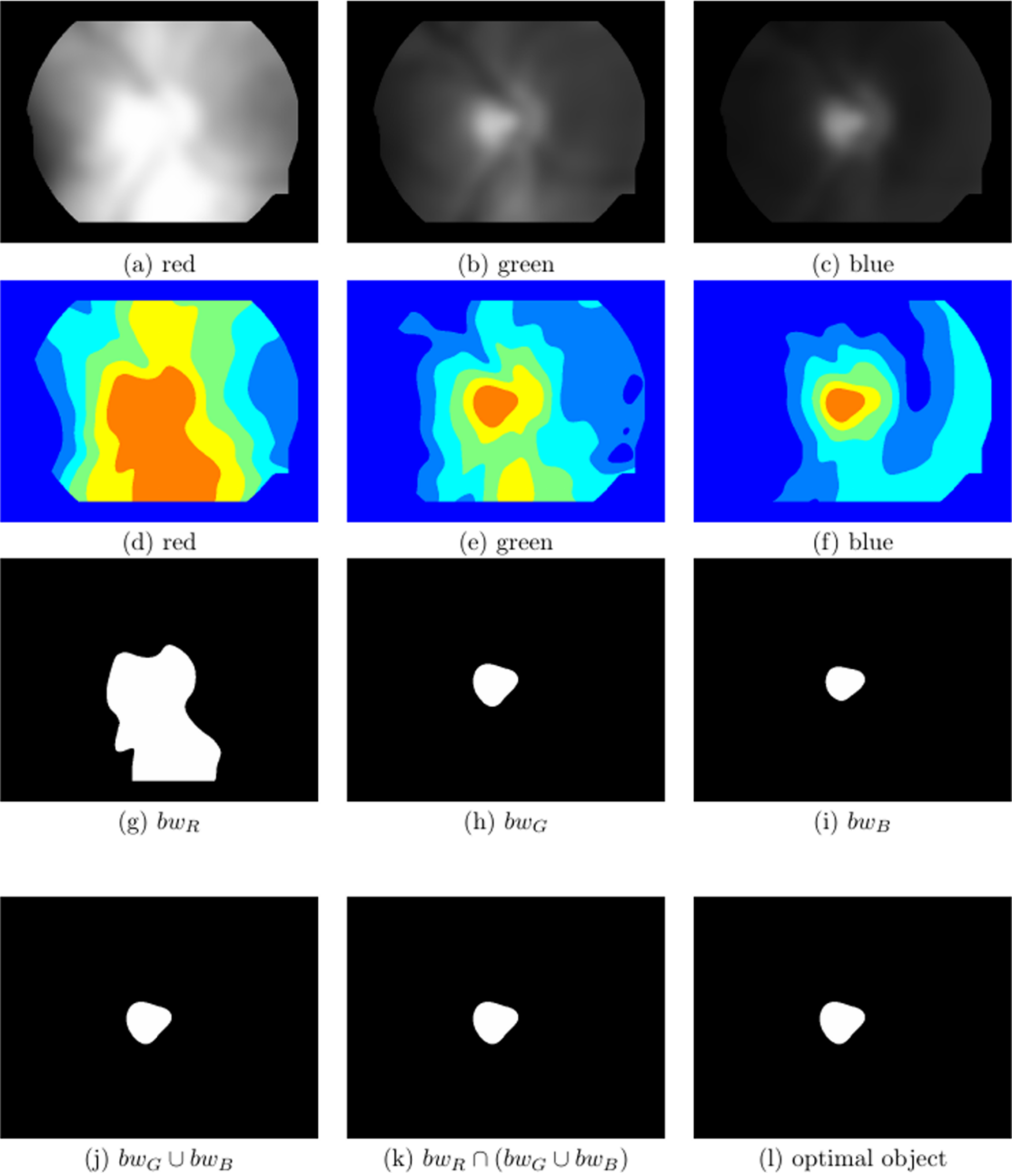
Visual multispectral analysis of a saturated image. (A–C) Normalised and Gaussian filtered images from the red, green and blue channels, respectively, (D–F) show the classes after multithreshold processing from the image above, for *k* = 5, (G–I) are the maximum class defined as bw_R_, bw_G_ and bw_B_, respectively, (J) bw_G_ ∪ bw_B_, (K) bw_R_ ∩ (bw_G_ ∪ bw_B_) and (L) optimal selected object.

The purpose of this filter is to retain objects that are the approximate size of the desired OD and blur or eliminate other smaller objects such as blood vessels, exudates and/or haemorrhages, see Figs. 4A–4C and 5A–5C, unsaturated and saturated images, respectively.

#### Multispectral analysis

A division of classes is performed in each of the channels of the visual spectrum R, G and B, using Otsu multi-level thresholding (Otsu, 1979). Otsu’s method consists of obtaining the difference between the variances of two classes and finding the threshold that minimises this difference. To extend this idea for multilevel thresholding considering ‘*k*’ thresholds, it separates the original image into *k* + 1 classes. It is necessary to compute the *k* variances and their respective probabilities and means. This objective function can be written for multiple thresholds as follows:

(3) $$J\left( {{\rm{TH}}} \right) = \max \left( {{{\rm{\sigma }}^{{2^c}}}\left( {{\rm{TH}}} \right)} \right),\quad 0 \le {\rm{t}}{{\rm{h}}_i} \le L - 1,i = 1,2, \ldots ,k$$

where $c \in \left\{ {{\rm{r}},{\rm{g}},{\rm{b}}} \right\}$ and *L* is the intensity level from each component, *c*, of a RGB (red = r, green = g and blue = b) image, TH = [th_1_, th_2_, . . ., th_*k*_] is a vector containing thresholds and the variances are computed through:

(4) $${{\rm \sigma }^{{2^c}}} = \sum\limits_{i = 1}^k {{\rm \omega }_i^c} {\left( {{\rm \mu }_i^c - {\rm \mu} _T^c} \right)^2}$$

where, *i* represents the *i*th class, ${\rm{\omega }}_i^c$ and ${\rm{\mu }}_i^c$ are, respectively, the probability of occurrence and the mean of a class. $${\rm{\mu }}_T^c = {\rm{\omega }}_1^c{\rm{\mu }}_1^c + {\rm{\omega }}_2^c{\rm{\mu }}_2^c + \ldots + {\rm{\omega }}_k^c{\rm{\mu }}_k^c$$; and ${\rm{\omega }}_1^c + {\rm{\omega }}_2^c + \ldots + {\rm{\omega }}_k^c = 1$. We found that *k* = 5 is adequate for all the images we analysed. The problem of finding the multiple thresholds, is now reduced to finding the intensity levels (TH) that maximises Eq. (3), via the simplex search-based optimisation method of Lagarias et al. (1998) (See Figs. 4D–4F and 5D–5F).

Using the *k*th threshold of the TH vector, the maximum intensity class in each of the channels is extracted, as bw_R_, bw_G_ and bw_B_, Figs. 4G–4I and 5G–5I. Finally, the information of these binary images is combined in the following way:

(5) $${\rm{b}}{{\rm{w}}_{{\rm{out}}}} = {\rm{b}}{{\rm{w}}_{\rm{R}}} \cap \left( {{\rm{b}}{{\rm{w}}_{\rm{G}}} \cup {\rm{b}}{{\rm{w}}_{\rm{B}}}} \right)$$

Typically, in a well contrasted image (Figs. 2A–2C), both the OD and exudates appear clearly in the red channel. In the green channel these two patterns appear but, with less intensity, especially the OD. While in the blue channel these features are not easily discernible.

When the green and blue channels are normalised, the most intense pattern in both is usually the OD, so the union of these two channels $\left( {{\rm{b}}{{\rm{w}}_{\rm{G}}} \cup {\rm{b}}{{\rm{w}}_{\rm{B}}}} \right)$, is almost certain to have the OD pattern present, see Figs. 4J and 5J. Sometimes there are other ‘artefacts’ that compete with the OD, for example the edges of the FOV due to an excess of illumination (see Fig. 4J).

When the red channel is saturated (Figs. 3A–3C), the object with high intensity will have a much larger area than the OD sought, which will usually be immersed in it. Hence, an intersection of bw_R_ with the previous union, Figs. 4K and 5K, will give a high probability of finding the OD that appears in the three channels and eliminate other artefacts such as exudates or excess of illumination at the edges of the FOV, that are less intense than the OD. It is important to emphasise that the identification of the OD does not need to be precise at this stage, it is only necessary to identify a region which is coincident with the OD without necessarily being congruent.

#### Morphological analysis

Most of the time, Eq. (5) results is a single object, however, in some cases more than one object is obtained, either because the OD appears fragmented into two sections (e.g. by a blood vessel passing through it) or because some other sufficiently intense patterns (such exudates or luminous artefacts) are also found in some of the channels (typically in the green or blue).

In this case mathematical morphology is used to analyse the shapes of the objects (see Fig. 4K). The object that has

(6) $${\rm{Roundness}} = {{4{\rm{\pi Area}}} \over {{\rm{Perimete}}{{\rm{r}}^2}}} \ge 0.6;$$

will be selected. If more than one object fills this criterion, then the selected object will be the one whose difference between its equivalent radius and *R_a_* is the smallest.

(7) $$\min \left( {\left| {\sqrt {{{{\rm{Are}}{{\rm{a}}_i}} \over {\rm{\pi }}}} - {R_a}} \right|} \right);$$

Once a single object has been obtained, the coordinates of its centroid will be the nominal centre used as a reference to obtain the ROI for use in the next phase of the analysis (see Figs. 4L and 5L).

### OD detection (fine scaling)

#### Pre-processing

Using the three normalised channels obtained in the coarse scaling phase (Figs. 2D–2F and 3D–3F), the blood vessels are initially removed using a mathematical morphological operation known as a *closing* (Serra, 1982).

Closing is defined as dilation followed by an erosion of an image *A* using the same structuring element, se:

(8) $$A \bullet {\rm{se = (A}} \oplus {\rm{se)}} \ominus {\rm{se;}}$$

where ⊕ and ⊖ denote dilation and erosion, respectively. Closing is usually used to remove small gaps. It selectively fills in background regions of an image if a suitable structuring element can be found that fits inside regions that are to be preserved, but does not fit inside regions that are to be removed.

We can think of blood vessels being ‘gaps’ in the retinal background. The optimal structural element to remove the blood vessels with this morphological operation is a disk-shape, whose radius must be defined to be a bit larger than the width of the largest vessel in the image.

Anatomically, the average ratio of the diameter of the main retinal blood vessels to the diameter of the OD, is approximately 0.15 and does not vary substantially between subjects (Jonas, Gusek & Naumann, 1988). Using this relation, we define the size of the structural element, se as:

(9) $${\rm{se}} = 0.15{R_a};$$

Finally, a small average filter of size 3 × 3 pixels is applied to blur any remaining edges of the vessels (Figs. 2G–2I and 3G–3I).

#### Information content (Shannon entropy)

**Figure 6:**
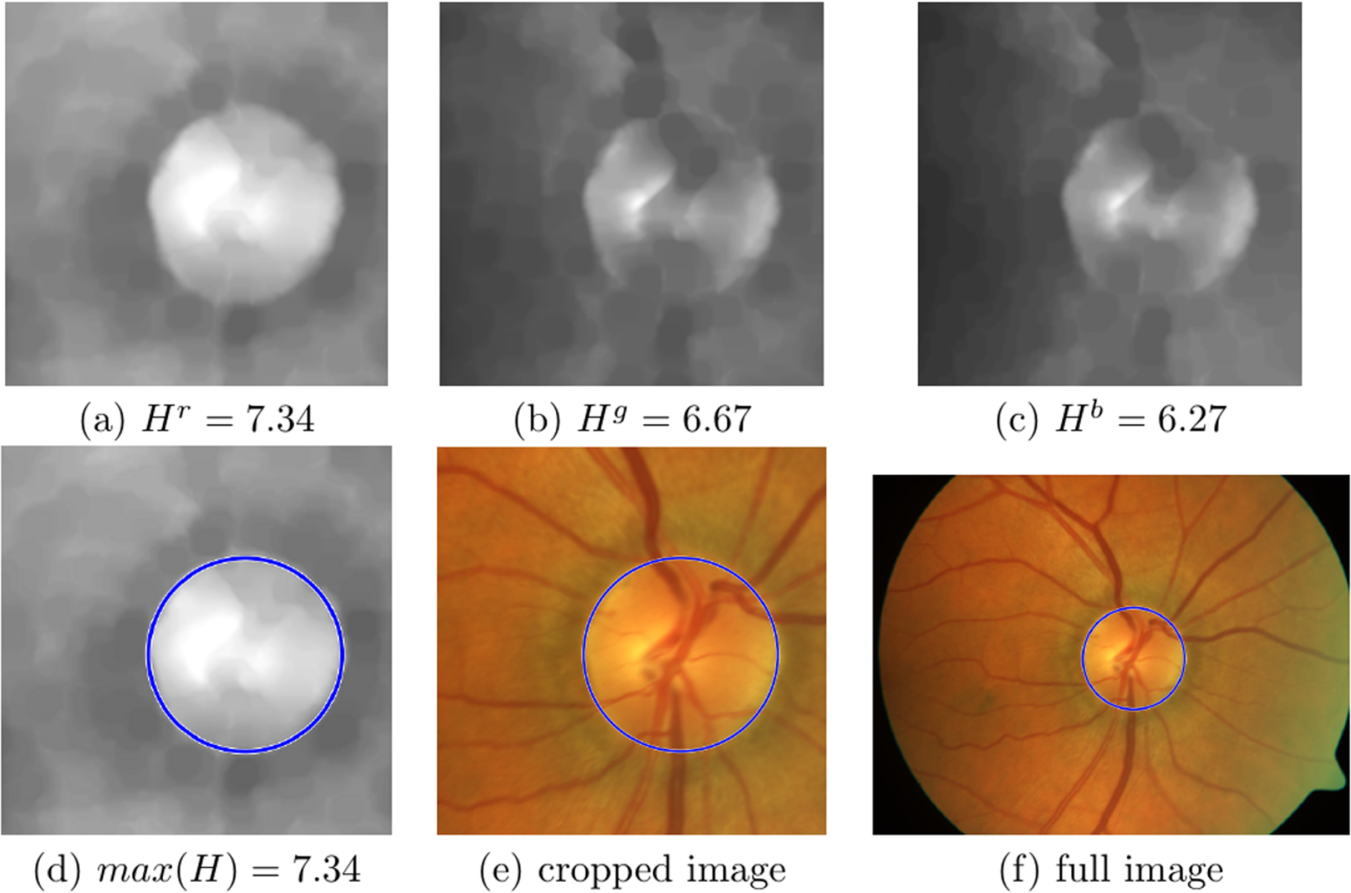
Information content analysis and circular Hough transform (CHT) in an image where red channel is not saturated. (A–C) The cropped unsaturated images with the vessels removed from the red, green and blue channels. Entropy values are: H^r^ = 7.34, H^g^ = 6.67 and H^b^ = 6.27, respectively, (D) the maximum entropy is found in the red channel, with the CHT circle marked in blue, (E) original cropped colour with the CHT circle marked in blue and (F) full colour image with the CHT circle marked in blue.

**Figure 7:**
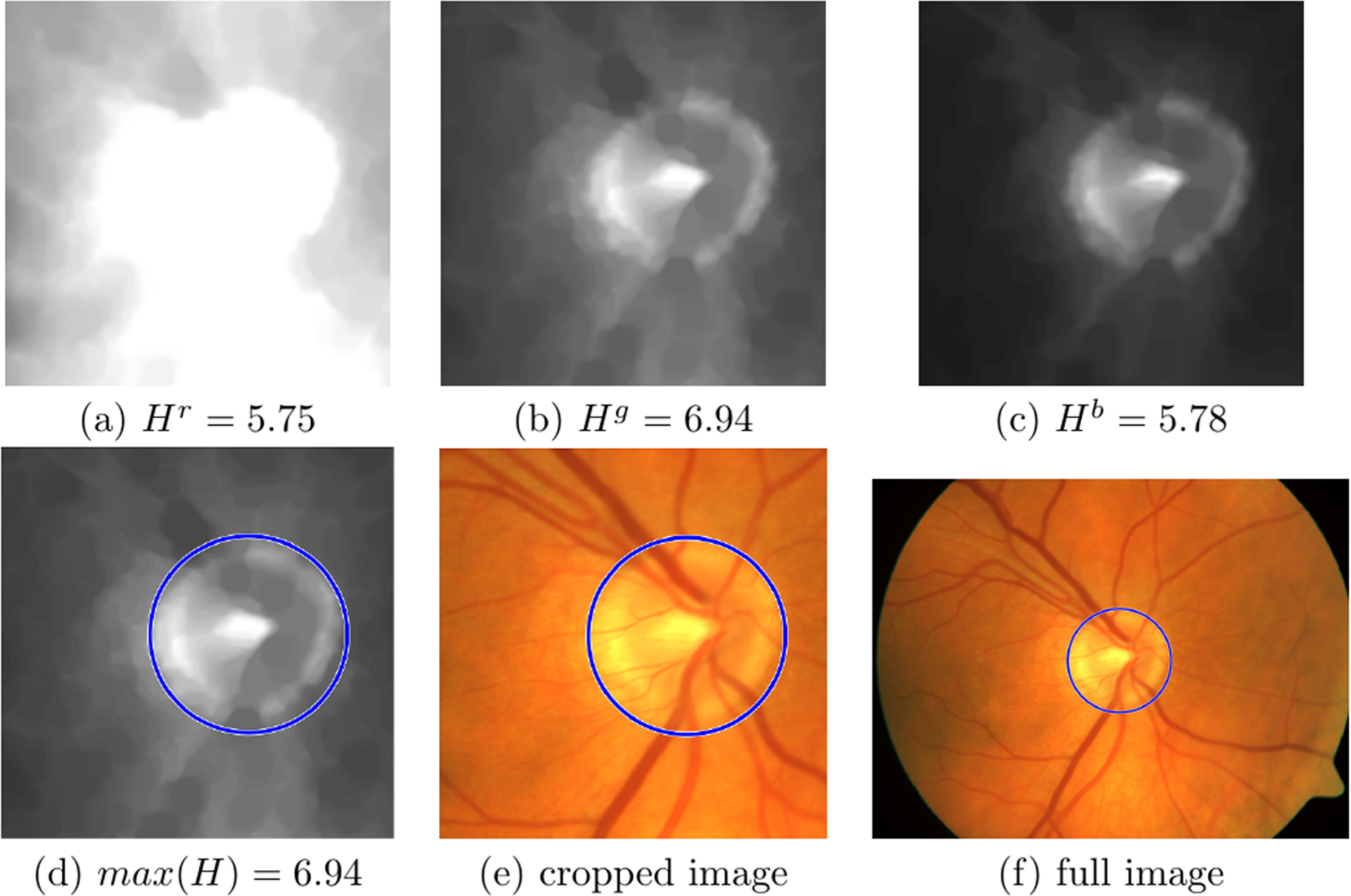
Information content analysis and circular Hough transform (CHT) in an image where red channel is saturated. (A–C) The cropped saturated images with the vessels removed from the red, green and blue channels. Entropy values are: H^r^ = 5.75, H^g^ = 6.94 and H^b^ = 5.78, respectively, (D) the maximum entropy is found in the green channel, with the CHT circle marked in blue, (E) original cropped colour with the CHT circle marked in blue and (F) Full colour image with the CHT circle marked in blue.

Each of these images are cropped to a square of size 2*R_a_* × 2*R_a_*, using the coordinates of the centroid found in the coarse scaling phase as a central reference (Figs. 6A–6C and 7A–7C).

The next stage in the analysis is to determine which of the channels should be used to search for the OD. If the image is well contrasted (Figs. 2A–2C), the most convenient channel would be red, however, when the red image is saturated (Figs. 3A–3C) this is not a good option. To be able to select the optimal channel automatically, the Shannon entropy of the ROI in each channel is calculated (Shannon, 1948):

(10) $${H^c} = - \sum\limits_{i = 1}^L {p_i^c} {\log _2}\left( {p_i^c} \right)$$

where *c* is the channel, $p_i^c$ is the normalised histogram or the probability distribution function of the channel *c* image and *L* the number of grey levels in the dynamic range.

The Shannon entropy is generally identified with the information content of the image. Therefore, we search for the OD in the channel where the entropy is greatest, ${\rm{max}}\left({{H^c}} \right)$. Figures 6A–6C shows the three channels of the well contrasted colour image where the entropy values for the red, green and blue channel are: **H^r^ = 7.34**, H^g^ = 6.67 and H^b^ = 6.27, respectively; here the maximum is in the red channel. Figures 7A–7C show the three channels of a saturated colour image with entropy values for red, green and blue channel: H^r^ = 5.75, **H^g^ = 6.94** and H^b^ = 5.78, respectively, in this case the maximum is in the green channel.

#### OD detection by Hough transform (localisation and delineation)

To find the nominally circular OD present in the selected channel we use the CHT (Yuen et al., 1990). The following discussion is based on (MathWorks, 2018):

1. Estimation of the centre. High-gradient foreground pixels are designated as candidate pixels and are allowed to ‘vote’ in the accumulator array. The candidate pixels vote in a pattern around them that forms a full circle of a fixed radius. The votes of the candidate pixels that belong to an image circle tend to accumulate in the container of the accumulator matrix corresponding to the centre of the circle. Therefore, the centre of the circle is estimated by detecting the peak in the accumulator array.
2. The radii are estimated explicitly using the centres of the circles estimated in step one, with a technique based on the calculation of radial histograms that generate a histogram of intensity as a function of the distance from the origin (centre); that is, they average the intensity on small rings and compute the average intensity of each ring.

The centre selected is the highest in the accumulator. The radius is defined as the first peak in the radial histogram.

A search range of circle sizes between [*R*_min_, *R*_max_] pixels is used (where *R*_min_ = 0.95*R_a_* and *R*_max_ = 1.3*R_a_*). This process finally returns the coordinates of the centre of the circle with the highest vote, and its respective radius. Figure 6D shows the circle found in the red channel of a well contrasted colour image, and Fig. 7D in the green channel of a saturated colour image. Figures 6E, 6F, 7E and 7F, show the respective results in the original colour images.

Finally, these two parameters are rescaled to the original image size of the initial input image (multiplying by a factor of 4 in this case). This ‘centre’ will be the one used to locate the measurement grid, and the circle found by the CHT will be the OD boundary delineation.

### Evaluation metrics and statistics

Two different evaluations are performed: (i) centre detection: normalised difference between OD centres using all three databases, and (ii) OD boundary delineation: contingency tables using the hand delineated files from the two public databases.

#### Centre location

In this application, we are interested in finding and evaluating the location of the OD centre to be able to fix the grid for further measurements and analysis (Fig. 1). As our local database of images (SABRE) has OD centres hand marked by the grader, we are able to compare distances between hand and automatic centres. The two public databases (DRIVE and MESSIDOR) have the OD area hand segmented, therefore, we compute their centroids, in order to make the same evaluation.

The metric takes the absolute difference between the centre marked by the grader, *C_m_* (ground truth—GT), and the centre found by the automatic method, *C_a_*, normalised by the size of the equivalent radius of the marked OD in pixels, *R_m_*. Since the OD was not marked in the SABRE database we normalised using the given a priori radius, *R_m_* = *R_a_*:

(11) $${R_m} = \sqrt {{{{A_{{\rm{OD}}}}} \over {\rm{\pi }}}} ;$$

(12) $$\Delta C = {{\left| {{C_m} - {C_a}} \right|} \over {{R_m}}};$$

where *A*_OD_ is the area of the marked OD in pixels. When Δ*C* ≥1 the automatic centre is outside the OD area defined by the grader, and therefore it is considered as a misdetection.

#### OD boundary delineation

Contingency tables are metrics based on true classifications. We will denote the results of our multispectral algorithm MS, and the hand-delineated as the GT. Contingency tables are built as follows: any pixel which is marked as OD in both GT and MS is a true positive (TP). Any pixel which is marked as non-OD in both GT and MS is a true negative (TN). Any pixel which is marked as OD in MS but non-OD in GT is a false positive (FP). The true positive rate (TPR) is established by dividing the number of TPs by the total number of OD pixels in GT. The false positive rate (FPR) is computed by dividing the number of FPs by the total number of non-OD in GT. A measurement of accuracy (Ac) can also be defined by the sum of TPs and TNs divided by the total number of pixels in the image. For a perfect area delineation, values of TPR and accuracy should be 1, whereas the FPR should be 0.

Finally, two other metrics that are commonly used to compare the results of area comparisons in the literature are: the overlap percentage (Abdullah, Fraz & Barman, 2016) and the Dice similarity index (Dice, 1945).

The overlap metric is defined as:

(13) $$\% {\rm{Overlap = }}{{{\rm{Area}}\left( {{\rm{GT}} \cap {\rm{MS}}} \right)} \over {{\rm{Area}}\left( {{\rm{GT}} \cup {\rm{MS}}} \right)}}$$

The Dice similarity index measures the similarity between the automatic delineated OD and the GT. The Dice index can be defined as two times the area of the intersection between the two delineations, divided by the sum of the areas of the two delineations, which is represented as:

(14) $${\rm{Dice}} = {{2*{\rm{Area}}\left( {{\rm{GT}}\ {{\mathop \cap \nolimits }}\ {\rm{MS}}} \right)} \over {{\rm{Area}}\left( {{\rm{GT}}} \right) + {\rm{Area}}\left( {{\rm{MS}}} \right)}}$$

To test the performance of the MS against using only the G or R channel, the mid *p*-value McNemar test was used (Fagerlan, Lydersen & Laake, 2013). This test is a nonparametric test to compare two classification models with binary responses. The null hypothesis tests whether model 1 (MS) and model 2 (G or R) have equal accuracy for predicting GT. *h* = 1 indicates rejection of the null hypothesis at the 5% significance level, while *h* = 0 indicates that the null hypothesis is not rejected at the 5% level.

This test is also used to compare rates of misclassification of pixels in individual images. The classification loss of each method, *e*_MS_ and *e*_G_ or *e*_R_, are the misclassification rate, a scalar in the interval [0, 1] representing the proportion of misclassified observations. Therefore, the McNemar test assess whether the accuracies of the classification models are different (*h*), and whether one classification model performs better than the other. We used Matlab R2018a (MathWorks, 2018) to calculate *h, p* and the misclassification rates, *e*_MS_ and *e*_G_ or *e*_R_.

## Results

### OD centre localisation

Table 1 compares MS to single channel analyses in terms of the number of images where the centre of the OD was outside of the OD determined by grader, N{**Δ*C* ≥ 1**} (see Eq. (12) for the definition of Δ*C*). For all databases MS performed better than analyses using a single channel: MS 2.6% (61/2,371), G 2.9% (68/2,371) and R 12.7% (301/2,371).

**Table 1:** OD localisation result.

	*N*{Δ*C ≥*1}	*N*_R_	*N*_G_	*N*_B_
SABRE (*N* = 1,131)				
MS	**30**	616	506	9
G	31	0	1,131	0
R	80	1,131	0	0
DRIVE (*N* = 40)				
MS	**1**	24	15	1
G	2	0	40	0
R	17	40	0	0
MESSIDOR (*N* = 1,200)				
MS	**30**	854	180	166
G	35	0	1,200	0
R	204	1,200	0	0

**Note:**

Results are shown for the three databases, the number in brackets, N, is the number of images in the database. The three rows per database, show the results for MS (multispectral), G (only green) and R (only red) tests. The first column (*N*{Δ*C* ≥ 1}) shows the number of images where the calculated centre of the OD lay outside the expert marked OD. The following three columns are the number of images where the search for the OD was carried in the red (*N*_R_), green (*N*_G_) and blue (*N*_B_) channels. The bold entries indicate the best performance.

Table 1 also includes information about how often the MS analysis used the information from the three channels in searching for the centre: R 63.0% (1,494/2,371), G 29.6% (701/2,371) and B 7.4% (176/2,371).

### OD boundary delineation

Table 2 shows the contingency tables (TPR, FPR, Ac, % Overlap and Dice index) derived using the MS or single channel analyses. For all metrics MS performed better than G or R channel in both public databases except for FPR in the DRIVE database. For the largest database, MESSIDOR, both the TPR and % Overlap metrics are significantly higher (approximately 10%) for the MS analysis.

**Table 2:** OD boundary delineation results.

	TPR	FPR	Ac	% Overlap	Dice
DRIVE (*N* = 40)					
MS	**0.882**	**0.003**	**0.993**	**81.0**	**0.881**
G	0.851	0.004	0.992	78.3	0.855
R	0.526	0.014	0.974	48.3	0.525
MESSIDOR (*N* = 1,200)					
MS	**0.876**	0.003	**0.994**	**80.2**	**0.875**
G	0.770	**0.001**	0.993	72.5	0.820
R	0.772	0.006	0.988	70.1	0.759

**Notes:**

Results are shown for the two public databases, DRIVE and MESSIDOR. The number in brackets, *N*, is the number of images in the database. The three rows per database, show the results for the MS (multispectral), G (only green) and R (only red) test. The columns show metrics derived from the contingency tables for the classification of pixels as OD versus the expert marked OD. TPR, the true positive rate; FPR, the false positive rate; Ac, the accuracy; % Overlap, the percentage of overlap; Dice, the Dice index. The bold entries indicate the best performance.

Table S1 shows the results of the McNemar test comparing the MS algorithm with each single channel method, first MS vs the G and second MS vs. R for both public databases. The first column gives the average *p*-value of images where *h* = 0 or *h* = 1. The following two columns give the average classification loss values of images where the misclassification rate was minimal for the MS algorithm (*e*_MS_) compared with the G or R algorithm (*e*_G_ or *e*_R_).

### Comparison to other work

Table 3 shows the contingency table from the DRIVE and Table 4 from the MESSIDOR databases using the MS method, as well as the metrics reported by other authors.

**Table 3:** Comparison of the delineation of the OD for DRIVE.

Method	TPR	FPR	Ac	% Overlap	Dice
MS	0.882	0.003	**0.993**	**80.95**	**0.881**
Roychowdhury et al. (2016)	0.878	**0.002**	0.991	80.67	–
Abdullah, Fraz & Barman (2016)	0.819	0.003	0.967	78.60	0.872
Basit & Fraz (2015)	**0.892**	0.008	0.986	61.88	–
Welfer, Scharcanski & Marinho (2013)	0.835	**0.002**	–	42.54	–
Morales et al. (2013)	0.854	0.006	0.990	71.63	0.817
Median [25th, 75th]	0.854 [0.831, 0.882]	0.003 [0.002, 0.007]	0.988 [0.977, 0.991]	71.63 [57.05, 79.12]	0.844 [0.817, 0.872]

**Notes:**

The contingency metrics calculated for the multispectral (MS) algorithm compared to the metrics published by other studies. Number of images is 40. The last row shows the median values and interquartile range [25th and 75th percentiles] taken from the published data. TPR, true positive rate; FPR, false positive rate; Ac, accuracy; % Overlap and Dice index. The bold entries indicate the best performance.

**Table 4:** Comparison of the delineation of the OD for MESSIDOR.

Method	TPR	FPR	Ac	% Overlap	Dice
MS	0.876	0.003	0.994	80.21	0.875
Sigut et al. (2017)	–	–	–	89.00	**0.939**
Zilly, Buhmann & Mahapatra (2017)	–	–	–	**90.00**	–
Abdullah, Fraz & Barman (2016)	0.895	**0.001**	**0.999**	87.93	0.934
Roychowdhury et al. (2016)	0.904	0.002	0.996	83.73	–
Lim et al. (2015)	–	–	–	88.80	–
Dashtbozorg, Mendonça & Campilho (2015)	**0.948**	**0.001**	**0.999**	88.59	0.937
Marin et al. (2015)	–	–	–	87.00	0.920
Giachetti, Ballerini & Trucco (2014)	–	–	–	87.90	–
Morales et al. (2013)	0.930	0.004	0.995	82.28	0.895
Cheng et al. (2013)	–	–	–	87.50	–
Median [25th, 75th]	0.917 [0.900, 0.939]	0.001 [0.001, 0.003]	0.997 [0.995, 0.999]	87.91 [87.0, 88.8]	0.934 [0.914, 0.938]

**Notes:**

The contingency metrics calculated for the multispectral (MS) algorithm compared to the metrics published by other studies. Number of images is 1,200. The last row shows the median values and interquartile range [25th and 75th percentiles] taken from the published data. TPR, true positive rate; FPR, false positive rate; Ac, accuracy; % Overlap and Dice index. The bold entries indicate the best performance.

In both tables the best results are marked in bold. The last row shows the median values and interquartile range taken from the published data. Although in the case of DRIVE, different GTs have been used in each publication and this comparison is, therefore, not straight-forward.

## Discussion

To test our hypothesis, we compared the performance of the MS algorithm to the performance of the same analysis restricted to either the R or the G channel. The results of these comparisons are shown in Tables 1 and 2.

Table 1 shows a number of images where the centre of the OD was found outside of the true nominal OD as the true centre as marked by an expert grader (Δ*C* ≥ 1). For all three databases the MS algorithm had fewer misdetections than the same analysis applied to either the R channel or the B channel. We also found that the percentage of misdetections by the MS algorithm was low, approximately 3% (61/2,371), over all three databases.

Table 1 also gives the number of images where the MS algorithm used the R, G and B channels in the determination of the centre of the OD, based on the Shannon entropy of the ROI in these channels. For all three databases the R channel was used most of the time, with the G channel used less than half as often. Interestingly in the MESSIDOR database where there were significantly more saturated images, the B channel was used nearly as often as the G channel. These results support our hypothesis about the utility of using all the spectral information in the image and highlight the difficulties that may arise if OD detection were limited to one colour channel.

Excluding the misdetections (Δ*C* < 1) the error in localisation was relatively small, Δ*C* = 0.08 [0.05, 0.13] (median and interquartile range [25th and 75th percentiles], corresponding to an absolute error of 0.14 mm assuming a disc diameter of 1.75 mm).

Our algorithm uses a CHT to detect the centre of the OD in the ROI determined by the initial stages of the MS algorithm. The CHT also returns a value for the most probable diameter of the circle which can be used to delineate the OD. Table 2 shows the contingency tables comparing the results of the MS algorithm to the effective radius of the OD marked by experts in the DRIVE and MESSIDOR databases. Because the expert ODs were not necessarily circular we have defined the equivalent radius as the radius of the circle with the same area as the expert OD (Eq. (11)) to be able to compare them to our necessarily circular ODs. With only one exception, the MS algorithm was superior to the same analysis applied to either the G channel or the B channel over all of the metrics. The one exception is the FPR which was lower when only the G channel was used for the images in the MESSIDOR database. The reasons for this single exception are unclear. Overall the results in Table 2 also support the hypothesis behind the MS algorithm.

As a further test of the MS hypothesis we compared the MS algorithm to analysis of either only the G or only the R channel using the McNemar mid *p*-value test for the classification of individual pixels in the delineation of the OD, for the DRIVE and MESSIDOR databases where expert marked ODs are available. The McNemar statistics provides a test of the null hypothesis that two methods give the same results when compared to the GT (taking *p* < 0.05 as significant), *h* = 0 means that the null hypothesis is supported and *h* = 1 means it is not. It also gives a rate of misclassification of pixels for the two methods, which indicates which of the competing methods is preferred when *h* = 1. Table S1 summarises the results of the McNemar test comparing MS to either of the single channel analyses.

The information from this test is limited in our case because it only assesses the ability of the two methods to correctly classify pixels in a single image as belonging to the OD or not. Looking at the results for all of the images, the average *p* values, where significant difference between the two methods was found (*h* = 1), were *p* < 0.003 for both comparisons (MS vs G and MS vs R) in both databases. Most significant for our purposes are the values of the classification loss, the average misclassification rate was minimum for the MS algorithm compare in all cases (*e*_MS_ < *e*_G_ or *e*_R_). Again the MS algorithm outperforms the single channel algorithms, adding further support for our hypothesis.

**Figure 8:**
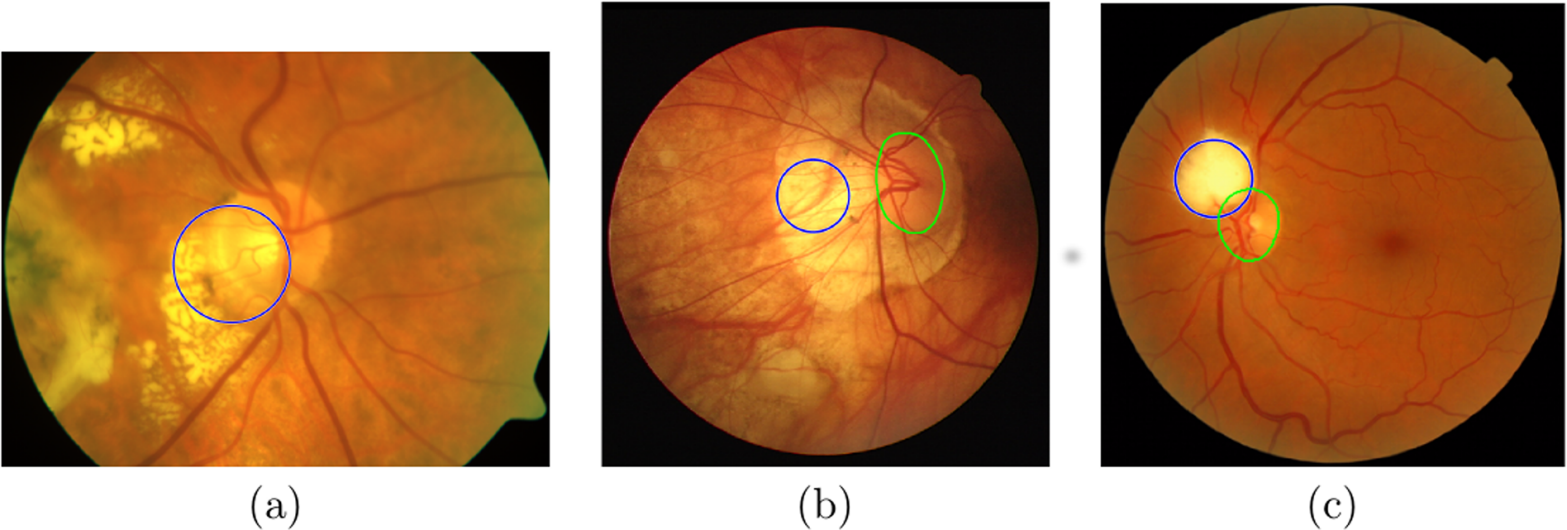
Example images where localisation failed. From (A) SABRE, (B) DRIVE and (C) MESSIDOR databases, where the automatic OD detection is shown in blue, (B) and (C) show the hand-delineated OD in green.

Figure 8 shows examples of misdetection of the OD in one image from each of the databases, where the automatic OD detection is shown in blue. Figures 8B and 8C show the hand-delineated OD in green. In each case a region of high intensity is found in all three channels, attributable to a lesion, leading to the selection of an incorrect ROI for further analysis. One strategy for overcoming this problem is to include information about the blood vessels (Martinez-Perez et al., 2007) in the selection of the ROI. This will be explored in future work.

The results discussed so far only address the performance of our algorithm for locating and delineating the OD when it uses all three colour channels compared to our algorithm applied to only a single colour channel. The results support our hypothesis that using all of the colour information is beneficial. We now turn to the comparison of the performance of our algorithm to other comparable studies published in the last 5 years. Since we do not have access to their detailed results, the comparison is made on the basis of the contingency tables published by these studies.

Table 3 shows the contingency tables for the classification of pixels in the OD for the DRIVE database. The best result for each metric is highlighted in bold. For this relatively small database (40 images) our MS algorithm does very well, being best in three of the five metrics.

Table 4 shows the same contingency tables for the larger MESSIDOR database (1,200 images). Our MS algorithm does not perform nearly as well, ranking last or next to last for every metric. Based on the data in this table the algorithm published by Dashtbozorg, Mendonça & Campilho (2015) is clearly the algorithm of choice for determining the area of the OD. Interestingly they used a combination of the R and G channels in their algorithm.

We have recorded data about the processing time to analyse an image using our MS algorithm. Since these data are so dependent on the computer, the size of the image and the programming environment, it is difficult to compare times in different studies. However, we note that our MS algorithm took an average of 0.32 s/image for the MESSIDOR database. The minimum time reported by the other studies listed in Table 4 was 1.28 s/image (Sigut et al., 2017), whereas Dashtbozorg, Mendonça & Campilho (2015) report 10.6 s/image. One reason for the speed of our MS algorithm is that we coarse scaled images that are greater than 1,000 pixels but for the DRIVE database no reduction was made and our algorithm still outperformed all of the published studies. It would interesting to see if trading processing time for image resolution would increase the accuracy of our localisation and delineation of the OD.

## Conclusions

The main hypothesis behind this work is that it should be beneficial to use all of the spectral information in a colour retinal image for the automatic detection and delineation of the OD even when some of that information may be saturated. We tested this by developing an algorithm that used information from all three channels (R, G and B) that could be used for a wide range of digital colour retinal images; different sizes, different FOV, OD or macular centered, different levels of saturation, taken with different cameras, settings and protocols (mydiatric and non-mydiatric). A novel feature of the algorithm is the use of the Shannon entropy of the images in the different colour channels to decide which channel should be used in the search for the OD.

Like all engineering problems the solution depends on a number of factors, some of them unquantifiable. As mentioned in the Introduction, the practical motivation for this work is to provide an algorithm that can assist in the analysis of the quantitative features of the retinal vasculature for large numbers of retinal images. We believe that no unsupervised computer algorithm can be used with complete confidence in medical studies; there will always be an image that defeats any algorithm. Our goal, instead, is to relieve the burden on the expert scanner by providing ‘most likely’ answers and flagging images where the expert needs to resolve difficult cases.

In conclusion, we provide evidence that there is an advantage in using information from all of the colour channels in a retinal image in locating and delineating the OD. Presumably, this should also be true in the computer analysis of the other features of the retina and the retinal vasculature, but that remains to be demonstrated.

## Supplemental Information

10.7717/peerj.7119/supp-1 Supplemental Information 1 Results of the McNemar test for DRIVE and MESSIDOR. Left section: MS vs G -multispectral vs. only green. Right section: MS vs R–multispectral vs. only red. Top: DRIVE, Bottom: MESSIDOR. h=0–null hypothesis is accepted, h=1–null hypothesis is rejected. First column: *p*–the average *p* value of images where h=0 or h=1, Second column: e_MS_–the average value for images where the misclassification rate is minimum for MS. Third column: e_G_ or e_R_–the average value for images where the misclassification rate is minimum for either G or R.

## Additional Information and Declarations

### Data Availability

The following information was supplied regarding data availability: The code and sample images are available at http://www.pcic.unam.mx/~elena/Research/ODdetection.html.
