# Supplementary material for: Automatic optic disc detection in colour fundus images by means of multispectral analysis and information content
Source: PeerJ. 2019 Jun 27;7:e7119. doi: 10.7717/peerj.7119 (PMC6599671; doi:10.7717/peerj.7119)
Supplement: Supplemental Information 1 — Left section: MS vs G -multispectral vs. only green. Right section: MS vs R–multispectral vs. only red. Top: DRIVE, Bottom: MESSIDOR. h=0–null hypothesis is accepted, h=1–null hypothesis is rejected. First column: p–the average p value of images where h=0 or h=1, Second column: eMS–the average value for images where the misclassification rate is minimum for MS. Third column: eG or eR–the average value for images where the misclassification rate is minimum for either G or R. [file peerj-07-7119-s001.docx]

|  | MS vs G | | |  | MS vs R | | |
| --- | --- | --- | --- | --- | --- | --- | --- |
| DRIVE | p | e_MS_ | e_G_ |  | p | e_MS_ | e_R_ |
| h=0 | 0.8318 | 0.0040 | 0.0040 |  | 0.9546 | 0.0024 | 0.0024 |
| h=1 | **0.0029** | **0.0045** | 0.0056 |  | **0.0001** | **0.0059** | 0.0287 |
| MESSIDOR |  | | | | | | |
| h=0 | 0.8583 | 0.0039 | 0.0039 |  | 0.9647 | 0.0016 | 0.0016 |
| h=1 | **0.0005** | **0.0022** | 0.0032 |  | **0.0005** | **0.0033** | 0.0085 |
